# House Poisons

**Published:** 1872-08

**Authors:** 


### HOUSE POISONS.

MANY persons have sickened and died after moving into new houses; others, after sleeping for a few nights, or even a single night in the "spare room" of a friend. A few years ago four children in one family sickened and died, one after another. In 1860, a woman sickened in Boston, manifesting all the symptoms of having been poisoned; she recovered to a certain extent, but never regained her health. In the case of the four children, the paper on the wall was found to contain three grains of arsenic in every square foot; in the case of the woman, a removal of the paper on the wall was followed with improvement in her health.

In all cases of pining sickness, when there is no appreciable reason for it, two things ought to be promptly done: change the room and the water; live all the time in an apartment without paper on the walls, or curtains about the windows, or any green color in the carpets; in addition, use water which is obtained from the roof of the house, and no other; or obtain water which is at least half a mile away, from a spring or well, many feet higher than the usual supplies, because the water may be poisoned by the lead pipes in the house, or more likely by the drainage of barn-yards, pig-pens, hen-houses, and privies finding its way into the well or spring, lower down than those, which supplies the family. As to curtains, carpets, and wall paper having a green color, it may be regarded as a certainty that the color is produced by the use of arsenic; and the glazing material, of whatever color, is mainly composed of a poisonous preparation of lead.

Precaution should be taken to exclude all green candies, all green toys, all glazed materials, even visiting cards, for a little child died recently by chewing a visiting card; it had a sweetish taste, having a glaze made of sugar of lead. In a toy box of water colors, one block of green paint, weighing forty grains, contained ten grains of arsenic; the green in lamp shades contains a large amount of arsenic, as do also the green papers which envelop the bon-bons of the confectioner. A tarlatan dress contained eight grains of white arsenic to every square foot of the material. Chemists are of the opinion that the dust of the arsenic is detached from these various objects by the moving air, or by handling, and is thus taken directly into the lungs, thence introduced into the blood. If any material supposed to contain arsenic is put into a small amount of hartshorn, spirits of ammonia, the liquid will have a bluish tint if arsenic is present; if further proof is desired, pour a little of this bluish liquid on crystals of nitrate of silver; if arsenic is present, there will be a yellowish deposit on the crystals.

But these things are not new, only disregarded; for a hundred years ago a law was passed in France forbidding the use of arsenic in making any colors for domestic uses; but its employment was so profitable in coloring many things, vases, artificial flowers, and the like, that the law was gradually more and more disregarded; and when its re-enactment was proposed, the shopkeepers rose in opposition, and declared it would ruin their business. Within a few years in England, a paper maker declared that he used four thousand pounds of arsenic every week in his workshops for the purpose of coloring and sizing.

A preparation for destroying vermin about houses is made largely of arsenic, called by various names; the most common is

SHEELES GREEN, being the arsenite of copper, the aceto-arsenite of copper, or

SHEINFUST GREEN, all dangerous to health and life, and should be sedulously excluded from every dwelling-house.

